# Efficient Estimation of CFO-Affected OFDM BER Floor in Small Cells with Resource-Limited IoT End-Points

**DOI:** 10.3390/s20133747

**Published:** 2020-07-04

**Authors:** Adriana Lipovac, Vlatko Lipovac, Borivoj Modlic

**Affiliations:** 1 Department of Electrical Engineering and Computing, University of Dubrovnik, 20000 Dubrovnik, Croatia; vlatko.lipovac@unidu.hr; 2 Faculty of Electrical Engineering and Computing, University of Zagreb, 10000 Zagreb, Croatia; borivoj.modlic@fer.hr

**Keywords:** LTE, IoT, end-point, BER estimation, time-sampling, BER abstraction

## Abstract

Contemporary wireless networks dramatically enhance data rates and latency to become a key enabler of massive communication among various low-cost devices of limited computational power, standardized by the Long-Term Evolution (LTE) downscaled derivations LTE-M or narrowband Internet of Things (NB IoT), in particular. Specifically, assessment of the physical-layer transmission performance is important for higher-layer protocols determining the extent of the potential error recovery escalation upwards the protocol stack. Thereby, it is needed that the end-points of low processing capacity most efficiently estimate the residual bit error rate (BER) solely determined by the main orthogonal frequency-division multiplexing (OFDM) impairment–carrier frequency offset (CFO), specifically in small cells, where the signal-to-noise ratio is large enough, as well as the OFDM symbol cyclic prefix, preventing inter-symbol interference. However, in contrast to earlier analytical models with computationally demanding estimation of BER from the phase deviation caused by CFO, in this paper, after identifying the optimal sample instant in a power delay profile, we abstract the CFO by equivalent time dispersion (i.e., by additional spreading of the power delay profile that would produce the same BER degradation as the CFO). The proposed BER estimation is verified by means of the industry-standard LTE software simulator.

## 1. Introduction

Deployment of the high-speed and low-latency wireless networks is under way to revolutionize the mission of Information and Communications Technology (ICT) in general. This is much more than just an upwards evolution of the fourth-generation (4G) Long-Term Evolution (LTE) broadband standard into the 5G New Radio (NR), but also a key enabler of Internet of Things (IoT) through the LTE’s downscaled derivations LTE-M and narrowband (NB) IoT, which regulate efficient interconnecting of many energy-sufficient low-cost devices, such as smart meters, various sensors, devices in vehicles, etc. [1,2,3]. Unfortunately, these are often computationally limited, specifically for assessing the physical-layer transmission performance, aimed to be used by higher-layer protocols to determine the extent of their involvement in the potential error recovery escalation upwards the stack [4].

As energy-efficient error control algorithms require reliable and accurate, but still simple and thus computationally efficient, estimation of transmission performance over various (sometimes mutually diverse) channels, it is quite a specific task to investigate [5,6].

Generally, digital data transmission is subject to symbol errors due to transmission media imperfections, such as time dispersion (mostly caused by multipath propagation) and additive noise, as well as other non-additive and non-linear imperfections on the part of equipment.

With this regard, the long lasting trend of replacing the bit error rate (BER) as a former sole qualifying transmission performance indicator, by the in-service measurable (and thus network operator friendly) block error rate (BLER), has finally ended by adopting the latter in LTE and 5G NR physical layer test standards [7,8].

Nevertheless, as the only parameter closing in the probability of a bit error, the BER still counts in the analysis, enabling estimation of BLER (by adopting a particular time distribution of erroneous bits within data blocks [7]), whose in-service direct measurement relies on identifying and counting erroneous blocks, which may be unreliable, especially when retransmission requests are rare, so that each failed block becomes more significant [9]. Moreover, it is the overall accuracy of BLER that effectively determines the so-called residual channel to the higher-layer protocols, and thus the cross-layer protocol stack design [7,8]. Specifically, if the connectionless User Datagram Protocol (UDP) is used at the transport layer for reduced overhead and increased throughput (i.e., “goodput“), inaccurate estimation of the physical layer transmission performance might either unnecessarily engage the application layer for this task (with even more overhead, the most expensive at this layer), or have the errors propagated through the stack up to the application itself.

With this regard, it is often needed to most efficiently estimate the orthogonal frequency-division multiplexing (OFDM) BER floor mainly affected by the OFDM-inherent transmission impairments–carrier frequency offset (CFO) and large peak-to-average power ratio (PAPR). However, for small cells and IoT end-points with limited computing power, we may consider PAPR as either insignificant (e.g., when the high-power amplifier (HPA) operates in its linear regime) or reduced by clipping or any other PAPR reduction method [10,11]. Moreover, in indoor or small-cell propagation environment, we may also assume the signal-to-noise ratio (SNR) to be quite large, as well as the OFDM symbol cyclic prefix (CP) guard time to be much longer than expected channel delay spread, thus preventing inter-symbol interference (ISI) due to channel time dispersion.

This implies that, practically, CFO remains a very dominant influencer of BER in the environment of interest here.

Accordingly, in contrast to earlier proposed computationally demanding estimation of BER being determined mainly by residual CFO, in Section 2, the OFDM BER floor model is developed for small cells and IoT end-points with limited computing power. In Section 3, we abstract the CFO by equivalent additional time dispersion of the power delay profile, to the extent that would produce the same BER degradation as the CFO. The proposed model is verified in Section 4 by conventional link abstraction based on the additive white Gaussian noise (AWGN), as well as by means of the industry-standard software simulator. Conclusions are reviewed in Section 5.

## 2. Efficient Estimation of Residual OFDM BER for Small Cells

### 2.1. General Error Floor Due to Time Dispersion

Let us first review the OFDM BER floor for propagation environments ranging from indoor to small-cell outdoor, where mostly strong signals and thus large SNRs can be expected in various 4G/5G scenarios for residual channel estimation.

Consider an OFDM symbol $\sum_{m=1}^{M}s_{m}e^{j\varphi_{m}}\cdot e^{jm\cdot \frac{2\pi }{MT_{s}}\tau }$ at an arbitrary sampling instant *τ*, as comprising *M* original symbols $s_{m}e^{j\varphi_{m}}$ of duration *T*_S_ each. On the other hand, the multipath channel is represented by the sum of complex delta functions, each with a Rayleigh-weighted amplitude $A_{i}$, uniformly distributed phase, and certain delay $\tau_{i}$, i∈[1,2,…,N].

Then the OFDM BER floor for the gray-mapped *m*-quadrature amplitude modulation (*m*-QAM) formats applied in 4G/5G is [12]:

(1) $$\begin{array}{ll} BER= & \frac{k_{m}}{2\sqrt{\pi }}\cdot \left(\sqrt{W^{-}E\left[{\left(\frac{\Delta \tau^{-}}{T_{s}}\right)}^{2}\cdot \Delta s_{n/n+1}^{2}\right]}+\sqrt{W^{+}E\left[{\left(\frac{\Delta \tau^{+}}{T_{s}}\right)}^{2}\cdot \Delta s_{n-1/n}^{2}\right]}-\frac{\sqrt{W^{-}E\left[{\left(\frac{\Delta \tau^{-}}{T_{s}}\right)}^{2}\cdot \Delta s_{n/n+1}^{2}\right]}\cdot \sqrt{W^{+}E\left[{\left(\frac{\Delta \tau^{+}}{T_{s}}\right)}^{2}\cdot \Delta s_{n-1/n}^{2}\right]}}{\sqrt{W^{-}E\left[{\left(\frac{\Delta \tau^{-}}{T_{s}}\right)}^{2}\cdot \Delta s_{n/n+1}^{2}\right]}+\sqrt{W^{+}E\left[{\left(\frac{\Delta \tau^{+}}{T_{s}}\right)}^{2}\cdot \Delta s_{n-1/n}^{2}\right]}}\right);\\ & k_{m}=\{\begin{array}{c} 1.000;m=4\mathrm{for}4-\mathrm{QAM}\\ 0.750;m=16\mathrm{for}16-\mathrm{QAM}\\ \begin{array}{c} 0.583 \end{array};m=64\mathrm{for}64-\mathrm{QAM} \end{array} \end{array}$$

where $\Delta \tau_{i}^{-}$ and $\Delta \tau_{i}^{+}$:

(2) $$\Delta \tau_{i}^{-}=\tau -\tau_{i}^{-};\Delta \tau_{i}^{+}=\tau -\tau_{i}^{+}$$

are relative delays of the channel response impulses, with respect to τ that distinguishes the preceding (“−”) from the delayed (“+”) multipath echoes.

Furthermore, the normalized “−” and “+” rms delay spreads in Equation (1) are defined as:

(3) $$\sqrt{E\left[{\left(\frac{\Delta \tau_{}^{-}}{T_{s}}\right)}^{2}\right]}=\sqrt{\frac{\sum_{i=1}^{N^{-}}{\left(A_{i}^{-}\right)}^{2}{\left(\frac{\Delta \tau_{i}^{-}}{T_{s}}\right)}^{2}}{\sum_{i=1}^{N^{-}}{\left(A_{i}^{-}\right)}^{2}}};\sqrt{E\left[{\left(\frac{\Delta \tau_{}^{+}}{T_{s}}\right)}^{2}\right]}=\sqrt{\frac{\sum_{i=N^{-}+1}^{N}{\left(A_{i}^{+}\right)}^{2}{\left(\frac{\Delta \tau_{i}^{+}}{T_{s}}\right)}^{2}}{\sum_{i=1}^{N^{-}}{\left(A_{i}^{+}\right)}^{2}}}$$

respectively, whereas the mean aggregate “−” and “+” echoes’ powers, relative to the total mean power, $W=\sum_{i=1}^{N}A_{i}^{2}=1$, are:

(4) $$W_{}^{-}=\sum_{i=1}^{N^{-}}{\left(A_{i}^{-}\right)}^{2},W_{}^{+}=\sum_{i=N^{-}+1}^{N}{\left(A_{i}^{+}\right)}^{2};W_{}^{-}+W_{}^{+}=1$$

respectively, where $N^{-}$ denotes the count of preceding echoes (out of *N* overall) that, timewise, corresponds to the sampling instance.

Moreover, the variances of differences between the *n*-th and the (*n*+1)-th OFDM symbol in the observed data sequence, and between the (*n*−1)-th and the *n*-th OFDM symbol $E\left[\Delta s_{n/n+1}^{2}\right]$ and $E\left[\Delta s_{n-1/n}^{2}\right]$, respectively [12]:

(5) $$E\left[\Delta s_{n/n+1}^{2}\right]=\sum_{m=1}^{M}\frac{s_{m,n}^{2}+s_{m,n+1}^{2}}{M^{2}},\begin{array}{cc} & E\left[\Delta s_{n-1/n}^{2}\right]=\sum_{m=1}^{M}\frac{s_{m,n-1}^{2}+s_{m,n}^{2}}{M^{2}} \end{array}$$

can reasonably be expected to be small, as the difference between neighboring OFDM symbols does not change fast; rather it can be regarded as almost constant for the observed (*n*-indexed) triplet of symbols.

Moreover, as the coefficient *k*_m_ in Equation (1) is the only distinguishing BER factor of higher modulation schemes with respect to binary phase-shift keying (BPSK), this implies that we can substitute the value 1/*M* for BPSK variances in Equation (5) into Equation (1), which transforms the latter to:

(6) $$BER=\frac{k_{m}}{2\sqrt{\pi }}\cdot \left(\sqrt{W^{-}E\left[{\left(\frac{\Delta \tau_{}^{-}}{\sqrt{M}T_{s}}\right)}^{2}\right]}+\sqrt{W^{+}E\left[{\left(\frac{\Delta \tau_{}^{+}}{\sqrt{M}T_{s}}\right)}^{2}\right]}-\frac{\sqrt{W^{-}E\left[{\left(\frac{\Delta \tau_{}^{-}}{\sqrt{M}T_{s}}\right)}^{2}\right]}\cdot \sqrt{W^{+}E\left[{\left(\frac{\Delta \tau_{}^{+}}{\sqrt{M}T_{s}}\right)}^{2}\right]}}{\sqrt{W^{-}E\left[{\left(\frac{\Delta \tau_{}^{-}}{\sqrt{M}T_{s}}\right)}^{2}\right]}+\sqrt{W^{+}E\left[{\left(\frac{\Delta \tau_{}^{+}}{\sqrt{M}T_{s}}\right)}^{2}\right]}}\right)$$

The BER floor estimation given by Equation (6), does not necessarily presume applying CP (to mitigate the multipath), but can easily incorporate it in the model by taking into account just the index range in Equations (3) and (4), which is outside of the CP span [12].

### 2.2. Computationally Efficient BER Floor for Small Cells

Furthermore, for the power delay profile rms delay spread up to 50 ns, data transmission speed in the order of, say, 100 Mbit/s, and *M* equal to 2048, 4096,…, we can justifiably consider that:

(7) $$E\left[\frac{\Delta \tau_{}^{-}}{\sqrt{M}T_{s}}\right],E\left[\frac{\Delta \tau_{}^{+}}{\sqrt{M}T_{s}}\right]<<1$$

Whereas from Equation (4) it is obvious that: $W^{-},W^{+}<1$.

This implies that the distinct “−” and “+” rms delay spreads $\sqrt{W^{-}E\left[{\left(\frac{\Delta \tau_{}^{-}}{\sqrt{M}T_{s}}\right)}^{2}\right]}$ and $\sqrt{W^{+}E\left[{\left(\frac{\Delta \tau_{}^{+}}{\sqrt{M}T_{s}}\right)}^{2}\right]}$, respectively, are both much smaller than unity. Consequently, their product in the numerator of the subtrahend in Equation (6) is negligible with respect to their sum in the denominator, implying that the whole subtrahend can be neglected, so Equation (6) can be justifiably approximated as it follows (in simpler notation where: $E\left[x\right]=\bar{x}$):

(8) $$BER=\frac{k_{m}}{2\sqrt{\pi M}T_{s}}\cdot \left(\sqrt{W^{-}}\cdot \sqrt{\bar{{\left(\tau -\tau_{}^{-}\right)}^{2}}}+\sqrt{W^{+}}\cdot \sqrt{\bar{{\left(\tau -\tau_{}^{+}\right)}^{2}}}\right)$$

where Equation (2) is taken into account to emphasize the arbitrary sample delay τ as the variable now.

### 2.3. Optimal Sample Delay for Least BER Floor

The optimal sample time instant $\tau_{\mathrm{opt}}$ which minimizes the BER floor, can be found by deriving Equation (8):

(9) $$\frac{d}{d\tau }\left(\sqrt{W^{-}}\cdot \sqrt{\bar{{\left(\tau -\tau_{}^{-}\right)}^{2}}}+\sqrt{W^{+}}\cdot \sqrt{\bar{{\left(\tau -\tau_{}^{+}\right)}^{2}}}\right)=0$$

Tedious testing of the second derivation is not necessary to be presented here, as no maximal *P*(e) value exists, so the first derivation is sufficient with this regard.

Furthermore, let us preselect the expected optimal sampling region: $\tau_{i}^{}\left(N^{-}\right)<\tau <\tau_{i}^{}\left(N^{-}+1\right)$ of the power delay profile where the “borderline” value of $N^{-}$ between the “−“ and “+“ echoes mutually balances $W^{-}$ and $W^{+}$, and thus maximizes the numerator of the second term in Equations (1) and (6), and consequently minimizes BER.

Moreover, if (without much loss of accuracy) we consider that both $W^{-}$ and $W^{+}$ are independent of τ, then after making derivation in Equation (9) we obtain:

(10) $$\sqrt{W^{-}}\cdot \frac{\bar{\tau -\tau_{}^{-}}}{\sqrt{\bar{{\left(\tau -\tau_{}^{-}\right)}^{2}}}}+\sqrt{W^{+}}\cdot \frac{\bar{\tau -\tau_{}^{+}}}{\sqrt{\bar{{\left(\tau -\tau_{}^{+}\right)}^{2}}}}=0$$

Solving the implicitly expressed Equation (10) provides the optimal sample delay $\tau =\tau_{\mathrm{opt}}$.

Moreover, considering balanced “−” and “+” rms delay spreads, both denominators in Equation (10) take mutually close values, so it reduces to:

(11) $$\sqrt{W^{-}}\cdot \left(\tau -\bar{\tau_{}^{-}}\right)+\sqrt{W^{+}}\cdot \left(\tau -\bar{\tau_{}^{+}}\right)\approx 0$$

enabling a simple approximate explicit expression for the optimal sample time:

(12) $$\tau_{\mathrm{opt}}\approx \frac{\sqrt{W^{-}}\cdot \bar{\tau_{}^{-}}+\sqrt{W^{+}}\cdot \bar{\tau_{}^{+}}}{\sqrt{W^{-}}+\sqrt{W^{+}}}$$

As it is obvious from Equation (12), generally, the optimal sample delay is not at the mean delay:

(13) $$\bar{\tau }=\frac{W_{}^{-}\cdot \bar{\tau_{}^{-}}+W_{}^{+}\cdot \bar{\tau_{}^{+}}}{W_{}^{-}+W_{}^{+}}=W_{}^{-}\cdot \bar{\tau_{}^{-}}+W_{}^{+}\cdot \bar{\tau_{}^{+}}=\sum_{i=1}^{N}{\left(A_{i}^{}\right)}^{2}\tau_{i}^{}$$

Specifically, for symmetrical delay profiles, such as, for example, the two-delay model [9], it is: $W^{-}\approx W^{+}\approx 0.5$, and: $\bar{\tau_{}^{-}}=-\bar{\tau_{}^{+}}$, implying that Equations (12) and (13) are identical in that the optimal sample delay is at the centerline of the power delay profile.

Thereby, as the mean delay alike, $\tau_{\mathrm{opt}}$ is also explicit in Equation (12), and determined by interpretable, though somewhat modified, delay dispersion parameters (distinct for “−“ and “+” echoes of the power delay profile).

However, apart from the general BER floor expression in Equation (6) that is really rather complex, from the perspective of low-performance network end-points, even Equation (12) is still somewhat more demanding for implementation than Equation (13); specifically as sampling at the mean delay further simplifies Equation (8), so that the residual BER becomes proportional to delay variance:

(14) $$BER=\frac{k_{m}}{2\sqrt{\pi M}T_{s}}\cdot \sqrt{\bar{{\left(\tau -\bar{\tau }\right)}^{2}}}=\frac{k_{m}}{2\sqrt{\pi M}T_{s}}\cdot \sqrt{\mathrm{var}\left(\tau \right)}$$

This, as well the formal similarity between Equations (12) and (13), raise the question of near-optimality of the mean delay (as the simplest to “tune-in”) (i.e., what will be the BER penalty for choosing the mean delay in Equation (13) instead of the optimal sample time in Equation (12).

Looking analytically, we can comment that the weighting factors of “−“ and “+” mean delays in Equation (12) are “−“ and “+” normalized aggregate rms values of the profile impulses’ amplitudes, whereas the powers themselves have the weighting role in Equation (13). This implies somewhat greater sensitivity of the mean delay on the imbalance between the “−“ and “+” parameters, while in Equation (13) it is a bit smoother.

Moreover, if we observe the *i*-th echo of the received OFDM symbol:

(15) $${\hat{r}}_{i}=\sum_{m=1}^{M}s_{m}e^{j\varphi }\cdot e^{jm\cdot \frac{2\pi }{MT_{s}}\left({\bar{\tau }}_{}+\Delta \tau_{\mathrm{opt}}-\tau_{i}^{}\right)};i=1,2,\ldots N$$

which is not time-sampled at the optimal sampling instant $\tau =\tau_{\mathrm{opt}}$, but with the offset of $\Delta \tau_{\mathrm{opt}}$ of the mean delay $\bar{\tau }$ from $\tau_{\mathrm{opt}}$, it introduces the phase shift:

(16) $$\vartheta_{i}^{}=m\cdot \frac{2\pi }{MT_{s}}\cdot \Delta \tau_{\mathrm{opt}}$$

and, consequently, the symbols’ constellation rotation, as well as scattering of symbol constellation spots, since Equation (16) depends on *m* (i.e., the rotation angle is echo-dependent).

**Figure 1:**
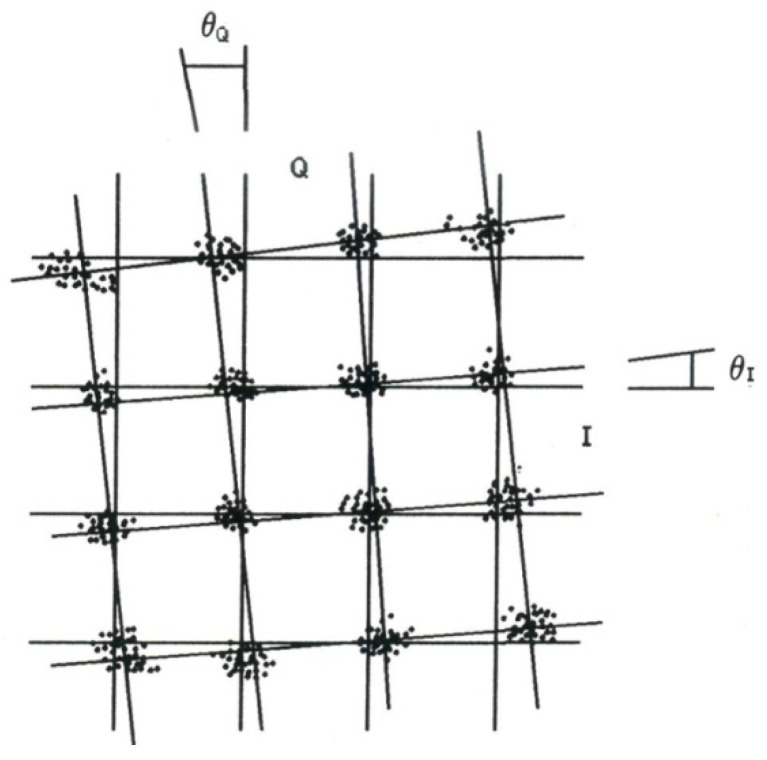
Carrier lock angle error [16].

This (rotation) outcome is similar to the carrier lock angle error [13,14,15] (while symbol spots scattering can be attributed to noise) being the arithmetic mean between the angles $\theta_{I}$ and $\theta_{Q}$ of the *I* and *Q* axis, respectively, and the cluster-fitting lines (Figure 1) [16].

The carrier lock error distorts the constellation effectively closing the eyes of *I* and *Q* components by degrading the equivalent SNR of the additive white Gaussian noise (AWGN), as well as the non-AWGN impairments abstracted by AWGN that would equally increase BER.

Moreover, under our assumption of a high SNR, which is quite realistic for small cells due to their short propagation paths [12], we expect that the OFDM BER floor is affected by time dispersion just to the extent for which the echo delays exceed the standard CP of 4.69 μs, which is very unlikely to occur in small cells [17].

This finally implies that the only remaining OFDM impairment with regard to BER floor is CFO, even if it was compensated (mostly in time domain), as a phase error may still reside (to be corrected, too, mostly in frequency domain).

## 3. Efficient OFDM BER Floor with Joint Near-Optimal Time Sampling and CFO

### 3.1. CFO Abstraction by AWGN

The impact of CFO on the BER floor (considered to be determined just by the CFO) can be modeled by conventional AWGN link abstraction, for example, as the PAPR was shown to affect the CFO-induced phase distortion $\Delta \Phi_{k\max}^{}$ of the *k*-th original (pre-OFDM) symbol, as a function of BER, with the CFO $\Delta f_{\mathrm{CFO}}$ as the parameter [18]. Inversing the relationship $\Delta \Phi_{k\max}^{}\left(BER\right)$, we obtain:

(17) $$BER\left(\Delta \Phi_{k\max}^{2}\right)\approx \frac{4}{\log_{2}M}\cdot Q\left[\sqrt{\frac{3}{M-1}}\cdot \frac{1}{10\cdot \log\left(10^{\frac{s_{k}^{2}}{30\cdot {\left(\Delta f_{\mathrm{CFO}}MT_{s}\right)}^{2}}\cdot \Delta \Phi_{k\max}^{2}}-10^{\frac{BACK-OFF}{10}}\right)}\right];1\leq k\leq M$$

where *Q*[·] is a Gaussian tail function. From Equation (18), it is obvious that its computational burden is quite demanding for network end-points with low processing capacity.

**Figure 2:**
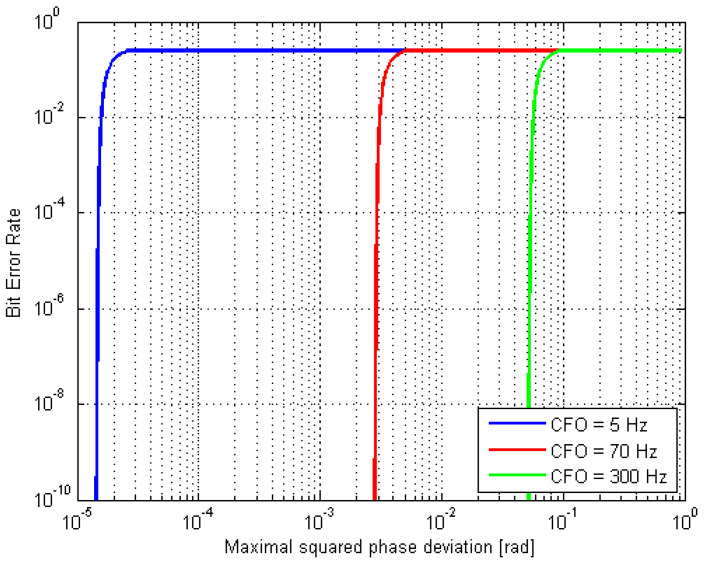
Bit error rate (BER) vs. maximal carrier frequency offset (CFO)-made phase deviation; 16 quadrature amplitude modulation (QAM), 10 dB high-power amplifier (HPA) back-off.

For HPA back-off of 10 dB, Equation (17) is graphed in Figure 2.

### 3.2. CFO Abstraction by Time Dispersion

If we observe the received OFDM symbol with offset $\Delta f_{\mathrm{CFO}}$ of the subcarrier frequency recovered at the receiver, with respect to the transmitted frequency $m\cdot \frac{2\pi }{MT_{s}}$, the *i*-th echo in Equation (15) of the received OFDM symbol transforms to:

(18) $${\hat{r}}_{i\mathrm{CFO}}=\sum_{m=1}^{M}s_{m}e^{j\varphi }\cdot e^{j\left(m\cdot \frac{2\pi }{MT_{s}}+\Delta f_{\mathrm{CFO}}\right)\cdot \left(\bar{\tau }+\Delta \tau_{\mathrm{opt}}-\tau_{i}^{}\right)};i=1,2,\ldots ,N$$

Now, the residual CFO being a non-AWGN impairment, can be abstracted not by conventional AWGN, but by, in this case more convenient, equivalent additional delay spread that would cause the same phase and thus BER floor degradation, described exclusively by time dispersion [12].

Accordingly, we modify Equation (19) considering the observed *i*-th echo CFO-free but with shifted sample time $\bar{\tau }$ for $\Delta \tau_{i\mathrm{CFO}}^{}$ that would introduce equal phase shift to that echo as it would to the CFO $\Delta f_{\mathrm{CFO}}$ [12]:

(19) $$\left(m\cdot \frac{2\pi }{MT_{s}}+\Delta f_{\mathrm{CFO}}\right)\cdot \left(\bar{\tau }+\Delta \tau_{\mathrm{opt}}-\tau_{i}^{}\right)=\left(m\cdot \frac{2\pi }{MT_{s}}\right)\cdot \left(\bar{\tau }+\Delta \tau_{\mathrm{opt}}+\Delta \tau_{i\mathrm{CFO}}-\tau_{i}^{}\right);i=1,2,\ldots ,N$$

From Equation (19), it implies that, for the actual echo, the additional delay $\Delta \tau_{i\mathrm{CFO}}$ can abstract the CFO frequency offset $\Delta f_{\mathrm{CFO}}$ under the following condition:

(20) $$\Delta \tau_{i\mathrm{CFO}}=\Delta f_{\mathrm{CFO}}\cdot \frac{MT_{s}}{2\pi m}\cdot \left(\bar{\tau }+\Delta \tau_{\mathrm{opt}}-\tau_{i}^{}\right)$$

As it can be seen from Equation (20), expectedly, the abstracting additional delay $\Delta \tau_{i\mathrm{CFO}}$ is directly proportional to the CFO and to the relative delay of the echo in subject to the mean delay (as $\Delta \tau_{\mathrm{opt}}<<$), and inversely to the subcarrier frequency (as lower sub channels are more CFO-sensitive).

Finally, the echoes’ delays of the equivalent CFO-inclusive power delay profile are linear with the non CFO-inclusive ones:

(21) $$\begin{array}{ll} \tau_{i\mathrm{EQ}} & =\tau_{i}+\Delta f_{\mathrm{CFO}}\cdot \frac{MT_{s}}{2\pi m}\cdot \left(\bar{\tau }+\Delta \tau_{\mathrm{opt}}-\tau_{i}^{}\right)=\\ & =\left(1-\Delta f_{\mathrm{CFO}}\cdot \frac{MT_{s}}{2\pi m}\right)\cdot \tau_{i}+\Delta f_{\mathrm{CFO}}\cdot \frac{MT_{s}}{2\pi m}\cdot \left(\bar{\tau }+\Delta \tau_{\mathrm{opt}}\right) \end{array}$$

## 4. Experimental Analysis

As the model is generally applicable to any OFDM-based wireless network, including the full-scale LTE (supported by our simulator) and its downscaled derivations LTE-M or NB IoT, we targeted no specific use case. Symbolically speaking, out of “mc^2^” (measure, compute and communicate) overall mission activities of cellular IoT networks, our model has to do with “c^2^” focusing on the second “c” (i.e., communicate), while taking care of the first one (compute) efficiently; meaning that the model applicability is not limited by any specific “m” scenario (i.e., use case) of OFDM-based wireless networks.

The efficient BER floor estimation model with near-optimal sampling at the mean delay of the power delay profile, as well as with CFO abstraction by time dispersion, was verified by comparing the results achieved two-fold: first by Equations (12)–(14) and (21), and then by software simulations.

### 4.1. Simulation Setup

**Figure 3:**
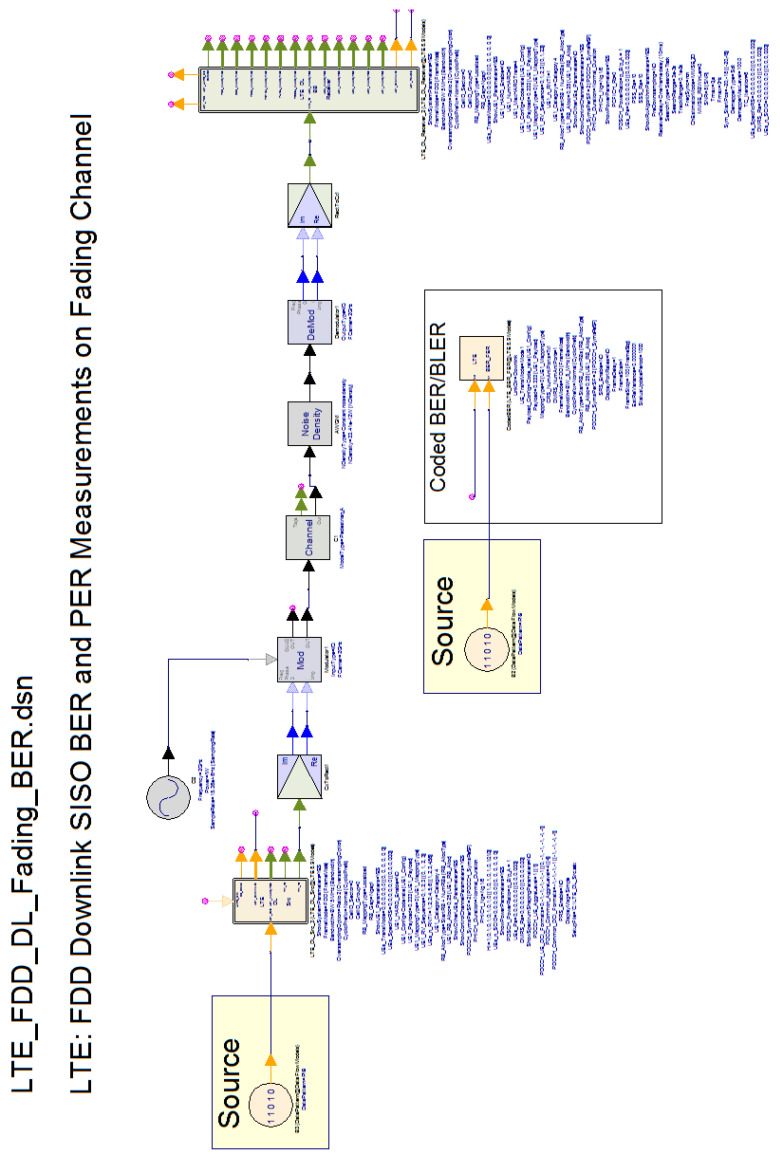
Long-Term Evolution (LTE) FDD downlink BER simulator setup.

We used the industry-standard, system-level software simulator SystemVue from Keysight Technologies, tailored for LTE (i.e., 5G NR numerology 0) physical layer tests [19]. Specifically, we configured it for measuring BER on the OFDM-based LTE Frequency-Division Duplex (FDD) downlink, with channel bandwidth of 5 MHz, packet switching, and 16 QAM exemplar modulation type [19]. We conducted the embedded framed BER tests on a specific Single-Input Single-Output (SISO) LTE downlink channel (PDSCH 1 (UE 1)), whereas the reference channels complied to TS 36.101, and the faded one to the definition in Annex B of TS 36.101 (Figure 3).

However, some embedded assumptions of the professional tool operation were not a good match for the analytical model verification. In this regard, we needed to disable the embedded CP data protection [19] (which we could only do by manipulating input data so as to mimic operation beyond the normal CP length of 4.69 μs), as well as disable the hybrid automatic repeat request (HARQ) protocol error control at physical (PHY) and Medium-Access Control (MAC) layers.

As the simulator setup provides sampling just at the very beginning of the delay profile with no other option, the only available means to simulate impact of various sample delay positions on BER was “nesting” progressive additional delays in the profile, while tracking BER reduction to the minimum.

**Figure 4:**
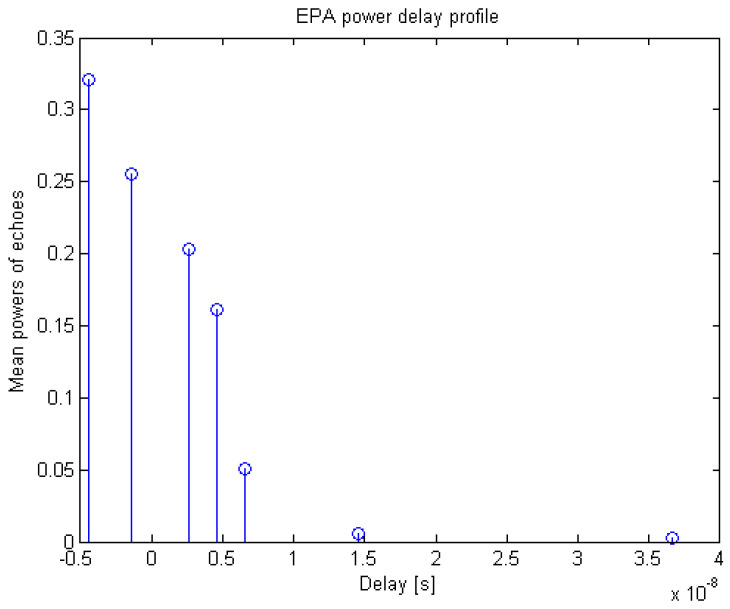
Extended pedestrian A (EPA) power delay profile.

**Figure 5:**
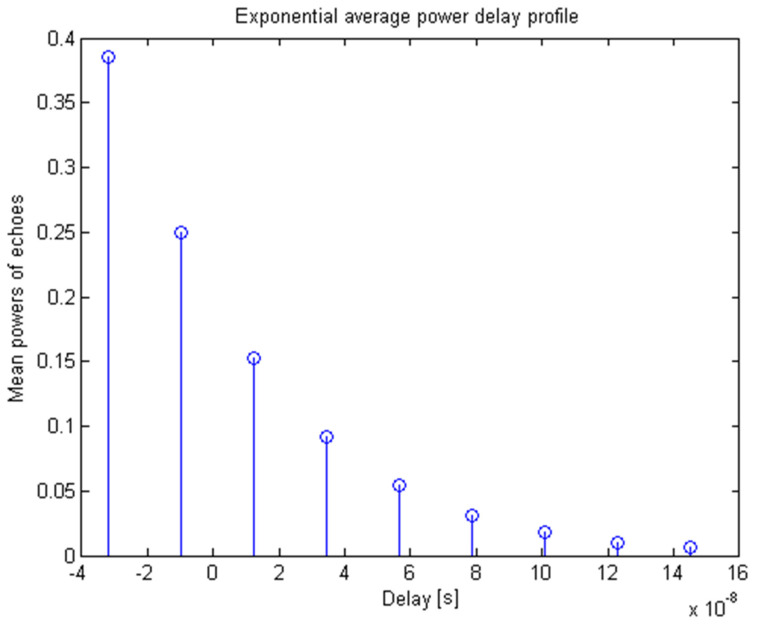
Exponential profile; rms delay spread $\sigma_{\tau }=2T_{S};$ $T_{S}=76\begin{array}{c} \mathrm{ns} \end{array}$.

We used two test power delay profiles, namely the extended pedestrian A (EPA) standard power delay profile model (Table 1, Figure 4) and the delay-limited exponential model (Figure 5), where for the latter, the a priori chosen probability *p* = 99% determines the maximal delay to be 4.6 times the rms delay spread, thus leaving just 1% of the corresponding delay-unlimited exponential profile to remain beyond the span of the limited one [19].

### 4.2. Verification of Efficient BER for Small Cells with Optimal and Near-Optimal Sampling

Firstly, we checked the accuracy (in terms of both BER and delay) of the optimal sample time $\tau_{\mathrm{opt}}$ estimated by Equation (12), with respect to the values obtained by simulations, where the latter was essentially based on the “sliding” process of trying all sample instants to identify the one, which minimizes the number of error occurrences.

**Figure 6:**
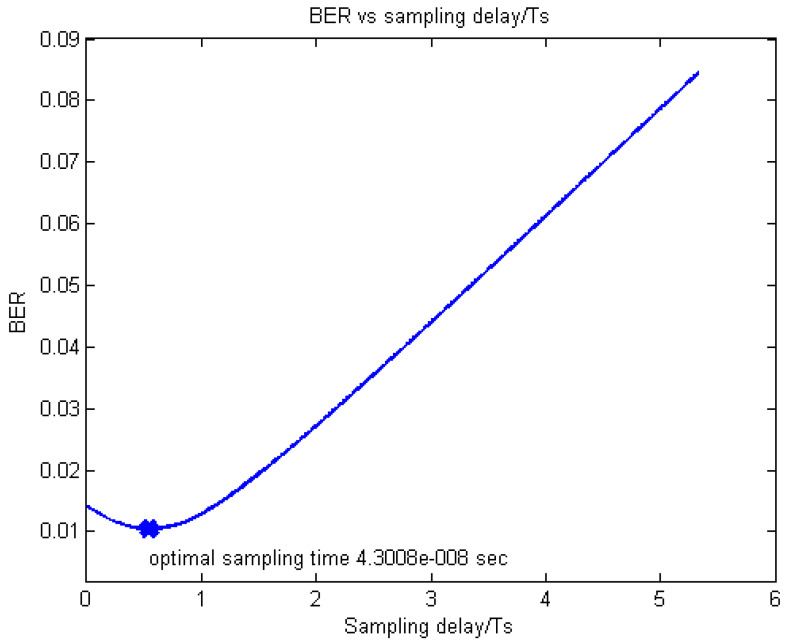
Estimated and simulated BER vs. (optimal) sample time for the EPA LTE FDD downlink.

**Figure 7:**
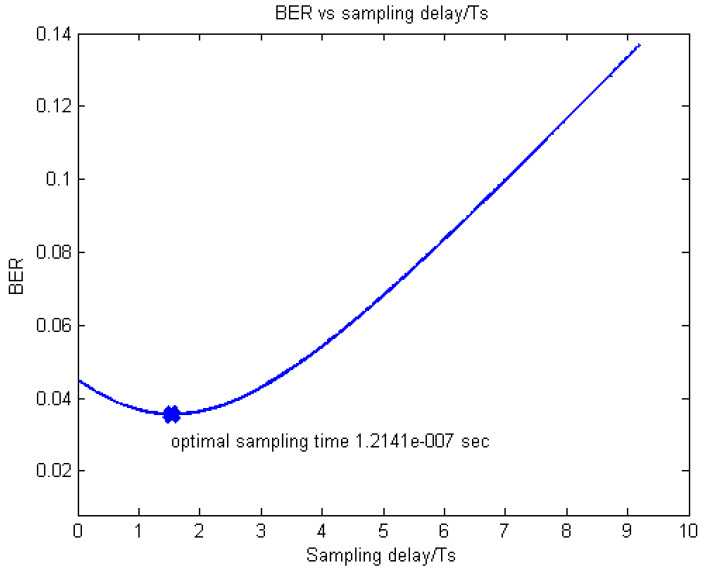
Estimated and simulated BER vs. (optimal) sample time for the exponential LTE FDD downlink.

The exemplar results obtained for the examined EPA and exponential profiles by simulations are presented in Figure 6 and Figure 7, respectively, where it can be seen that the obtained $\tau_{\mathrm{opt}}$ values are 4.3008 × 10^−8^ s and 1.2141 × 10^−7^ s, respectively.

On the other hand, the corresponding $\tau_{\mathrm{opt}}$ values, estimated according to Equation (12) are 4.2976 × 10^−8^ s and 1.2095 × 10^−7^ s, respectively, which is an excellent match to the figures obtained by the simulator.

After verifying Equation (12), we see in Figure 5 and Figure 6 that the curves around the minima are rather smooth and flat, so that we expect the optimal sample point to be quite close to the mean delay.

**Figure 8:**
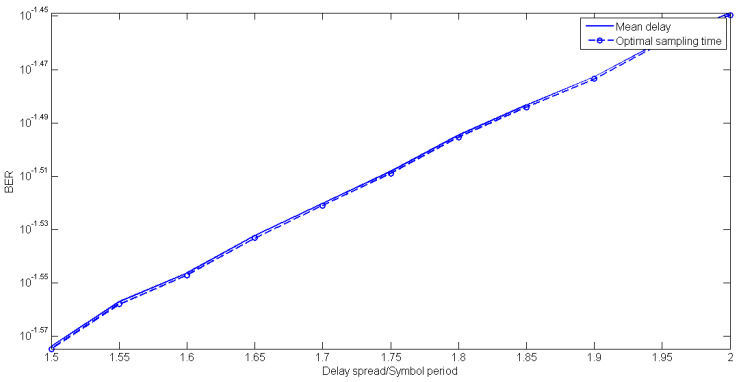
BER floor vs. rms delay spread for 16 QAM modulation for optimal and mean-delay sampling.

Indeed, sampling at the mean delay (Equation (13)), exhibits negligible worsening of BER with regard to the optimal sampling; just about 0.2% on average, for all three modulation types and both profiles, as evident in Figure 8, where the BER floor is graphed as a function of rms delay spread, adopting Equations (12) and (13). This finally verifies the use of the mean delay as the near-optimal sampling instant.

### 4.3. Verification of CFO Abstraction by Time Dispersion

Now, let us validate Equation (21) by accordingly modifying the EPA power delay profile to the equivalent CFO-inclusive ones for various CFO values, as presented in Table 1, Table 2, Table 3, Table 4, Table 5 and Table 6.

Thereby, the BER values coming out of both the ISI of the EPA channel and the five selected exemplar CFO values, are presented, for 16 QAM modulation type, in Table 7.

On the first look, the above BER values of the order of 1% seem quite high. However, these pertain to the case when the CP of 4.69 µs was not applied, otherwise the entire EPA power delay profile would be contained within the CP span, implying that BER = 0, whereas, for the exponential profile, BER would have taken very small values, closer to zero with “steeper” power delay profile. This paves the way to isolation of the impact of CFO alone on BER, once considering the CP applied.

Considering CFO as a random variable, which is statistically independent of other BER degrading impairments including ISI (due the EPA delay spread) in this case, we can justifiably consider their related error occurrences as mutually independent, too, and so additive. This further implies that we can calculate the CFO-exclusive BER values, as offsets with respect to the first value (for 0 Hz) in each column of Table 7.

This way obtained entries of Table 8 are BER penalties of the five selected exemplar CFO values, for 16 QAM modulation.

As we can see from Table 7 and Table 8, there is excellent matching between the estimated and the simulated CFO-induced BER floor values, which validates the CFO abstraction by the time dispersion model proposed here. Somewhat different values coming out of the AWGN abstraction (which is computationally demanding but we used it here as just another means of verification), are still in accordance with the ones coming out of the model as well as the simulator.

As we can see, only for the largest selected CFO of 5 kHz, supposedly acting on the very first subcarrier (*m* = 1) (i.e., for the worst-case CFO), the BER change with respect to the EPA profile is still minor, from 0.012969 to 0.023081.

## 5. Conclusions

In cellular IoT networks, there is a need for computationally non-demanding assessment of OFDM transmission performance that is either expressed by or related to the irreducible BER floor, which needs to be estimated by end-points of limited processing capacity, and directed upwards through the protocol stack to higher-layer protocols, thus influencing their error detection and correction actions.

The standard framework that the model targets is LTE, specifically its downscaled versions LTE-M and NB IoT, but its validity stretches to 5G NR as well.

Specifically, in indoor or small-cell outdoor environment, short propagation paths justify the assumption of high SNR, and enable the transmitter HPA to operate almost in a linear regime, thus effectively removing the excessive PAPR from the short list of the main BER influencers; quite alike as the time dispersion caused by inter-symbol interference was prevented by a large enough OFDM symbol cyclic prefix. This retains just the CFO (including the PAPR-induced additional CFO) as the sole OFDM impairment determining the BER floor in indoor or small-cell outdoor environment.

Furthermore, in contrast to earlier analytical models demanding considerable processing power for estimating BER, in this paper, after identifying the optimal sample instant in a power delay profile, we abstract the CFO by equivalent time dispersion that would produce the same BER degradation as the CFO. This finally provided the novel CFO-inclusive BER floor estimation proportional to delay variance of the power delay profile, as well as the CFO-exclusive BER estimation.

The proposed BER estimation is verified by the common AWGN link abstraction, and by means of the industry-standard software simulator in the LTE FDD downlink environment.

## Figures and Tables

**Table 1:** EPA delay profile [8]; mean= 0.2555, variance = 0.0613.

Tap	Delay (ns)	Power (dB)
1	0	0.0
2	30	−1.0
3	70	−2.0
4	90	−3.0
5	110	−8.0
6	190	−17.2
7	410	−20.8

**Table 2:** Modified EPA delay profile; CFO = 5 Hz; mean = 0.2556, variance = 0.0614.

Tap	Delay (ns)	Power (dB)
1	0	0.0
2	30.01	−1.0
3	70.02	−2.0
4	90.03	−3.0
5	110.04	−8.0
6	190.06	−17.2
7	410.14	−20.8

**Table 3:** Modified EPA delay profile; CFO = 70 Hz; mean = 0.2567, variance = 0.0619.

Tap	Delay (ns)	Power (dB)
1	0	0.0
2	30.14	−1.0
3	70.33	−2.0
4	90.42	−3.0
5	110.51	−8.0
6	190.91	−17.2
7	411.91	−20.8

**Table 4:** Modified EPA delay profile; CFO = 300 Hz; mean = 0.2606, variance = 0.0638.

Tap	Delay (ns)	Power (dB)
1	0	0.0
2	30.61	−1.0
3	71.41	−2.0
4	91.82	−3.0
5	112.23	−8.0
6	193.81	−17.2
7	418.22	−20.8

**Table 5:** Modified EPA delay profile; CFO = 1 kHz; mean = 0.2725, variance = 0.0698.

Tap	Delay (ns)	Power (dB)
1	0	0.0
2	32.01	−1.0
3	74.67	−2.0
4	96.11	−3.0
5	117.33	−8.0
6	202.67	−17.2
7	437.33	−20.8

**Table 6:** Modified EPA delay profile; CFO = 5 kHz; mean = 0.3406, variance = 0.1091.

Tap	Delay (ns)	Power (dB)
1	0	0.0
2	40.01	−1.0
3	93.33	−2.0
4	120.01	−3.0
5	146.67	−8.0
6	253.33	−17.2
7	546.67	−20.8

**Table 7:** Inter-symbol interference (ISI) and (time-dispersion-abstracted) CFO-based BER floor; 16 QAM, EPA profile.

CFO	BER Estimated (21)	BER Estimated (18)	BER Simulated
0 Hz (EPA)	1.2969 × 10^−2^	1.3141 × 10^−2^	1.2827 × 10^−2^
5 Hz	1.2990 × 10^−2^	1.3182 × 10^−2^	1.2901 × 10^−2^
70 Hz	1.3096 × 10^−2^	1.3244 × 10^−2^	1.2978 × 10^−2^
300 Hz	1.3498 × 10^−2^	1.3711 × 10^−2^	1.3415 × 10^−2^
1 kHz	1.4767 × 10^−2^	1.5013 × 10^−2^	1.4681 × 10^−2^
5 kHz	2.3081 × 10^−2^	2.3454 × 10^−2^	2.2901 × 10^−2^

**Table 8:** Time dispersion abstracted CFO-exclusive BER penalties; 16 QAM, EPA profile.

CFO	BER Estimated (21)	BER Estimated (18)	BER Simulated
0 Hz (EPA)	1.2969 × 10^−2^	1.3141 × 10^−2^	1.2827 × 10^−2^
5 Hz	0.0021 × 10^−2^	0.0041 × 10^−2^	0.0074 × 10^−2^
70 Hz	0.0127 × 10^−2^	0.0103 × 10^−2^	0.0151 × 10^−2^
300 Hz	0.0498 × 10^−2^	0.0570 × 10^−2^	0.0588 × 10^−2^
1 kHz	0.1798 × 10^−2^	0.1872 × 10^−2^	0.1854 × 10^−2^
5 kHz	1.0112 × 10^−2^	1.0313 × 10^−2^	1.0074 × 10^−2^
